# Sudden death associated with delayed cardiac rupture: case report and literature review

**DOI:** 10.3389/fcvm.2024.1355818

**Published:** 2024-04-12

**Authors:** Lopsong Tinzin, Xuefei Gao, Hui Li, Shuquan Zhao

**Affiliations:** ^1^ Sichuan Ding Cheng Judical Expertise Center, Chengdu, China; ^2^ Department of Pediatric Surgery and Urology-Andrology, First Moscow State Medical University named after Sechenov, Moscow, Russia; ^3^ Faculty of Forensic Medicine, Zhongshan School of Medicine, Sun Yat-Sen University, Guangzhou, China

**Keywords:** pathology, autopsy, delayed cardiac rupture, cardiac tamponade, case report

## Abstract

Cardiac injury plays a critical role in the process of thoracic trauma-related fatal outcomes. Historically, most patients who suffer a cardiac rupture typically die at the scene of occurrence or in the hospital, despite prompt medical intervention. Delayed cardiac rupture, although rare, may occur days after the initial injury and cause sudden unexpected death. Herein, we present the clinical details of a young man who suffered a chest stab injury and recovered well initially, but died days later due to delayed cardiac rupture. The forensic autopsy confirmed delayed cardiac rupture as the cause of death. We also reviewed previous similar reports to provide suggestions in such rare cases in clinical and forensic practice.

## Highlights

• We report a case of sudden death due to delayed cardiac rupture.
• Delayed cardiac rupture and tamponade post chest trauma were carefully reviewed.
• The present case and literature review could provide reference in such rare cases in forensic practice.

## Introduction

Thoracic trauma is the second-leading cause of morbidity and mortality and often involves cardiac injuries that can increase the mortality rate by 15 times (1). Given the varied definitions and diagnostic criteria of cardiac injury, its exact incidence is still unknown; however, previous studies have indicated that it ranges from 3% to 71% (2–4). Specifically, the incidence of cardiac rupture in chest trauma is reported to be 0.5% and is often associated with a lethal outcome (5). In 2023, Sessa et al. reviewed the published studies focusing on penetrating cardiac injury associated with firearm from 1990 to 2022, concluded that the morality of penetrating cardiac injury was affected by the location and severity of the heart injury, the interval between injury and medical intervention, the quantity of blood lost and presence of cardiac tamponade (6).

The presence of cardiac tamponade was common in the fatal cases indicating cardiac rupture is a medical emergency. Most patients die at the scene of occurrence without prompt medical intervention. By contrast, delayed cardiac rupture is a rare phenomenon that may cause sudden unexpected death in individuals with a history of chest trauma. It poses a significant threat to individuals who recover well at first after the initial chest injury in clinical practice. This complicates the link between the primary injury and subsequent fatal outcomes in forensic practice. However, the underlying mechanisms of delayed cardiac rupture are still debatable and require further investigation.

In this study, we present the clinical details of a young man who suffered a chest stab injury and recovered well initially, but died several days later because of delayed cardiac rupture, as confirmed by forensic autopsy.

## Case presentation

A 21-year-old man was stabbed in the left front chest with a folding fruit knife and was subsequently admitted to the hospital. Radiographic examination revealed left hemothorax. Closed thoracic drainage and blood-transfusion were performed, and the patient's condition stabilized. However, 5 days later, his condition suddenly deteriorated, and he died despite receiving timely medical intervention.

A forensic autopsy was performed one day later, which revealed bilateral hemothorax, and 800 ml and 500 ml blood were found in the left and right thoracic cavities, respectively. A 1.2-cm-long oblique strip with a sharp-edge wound was found on the right side of the pericardium and the middle of the right ventricular anterior wall. The pericardial cavity was filled with blood up to a volume of approximately 100 ml. A 1.0-cm-long wound with the same characteristics was found in the middle of the right ventricular anterior wall and pierced into the cardiac chamber.

**Figure 1:**
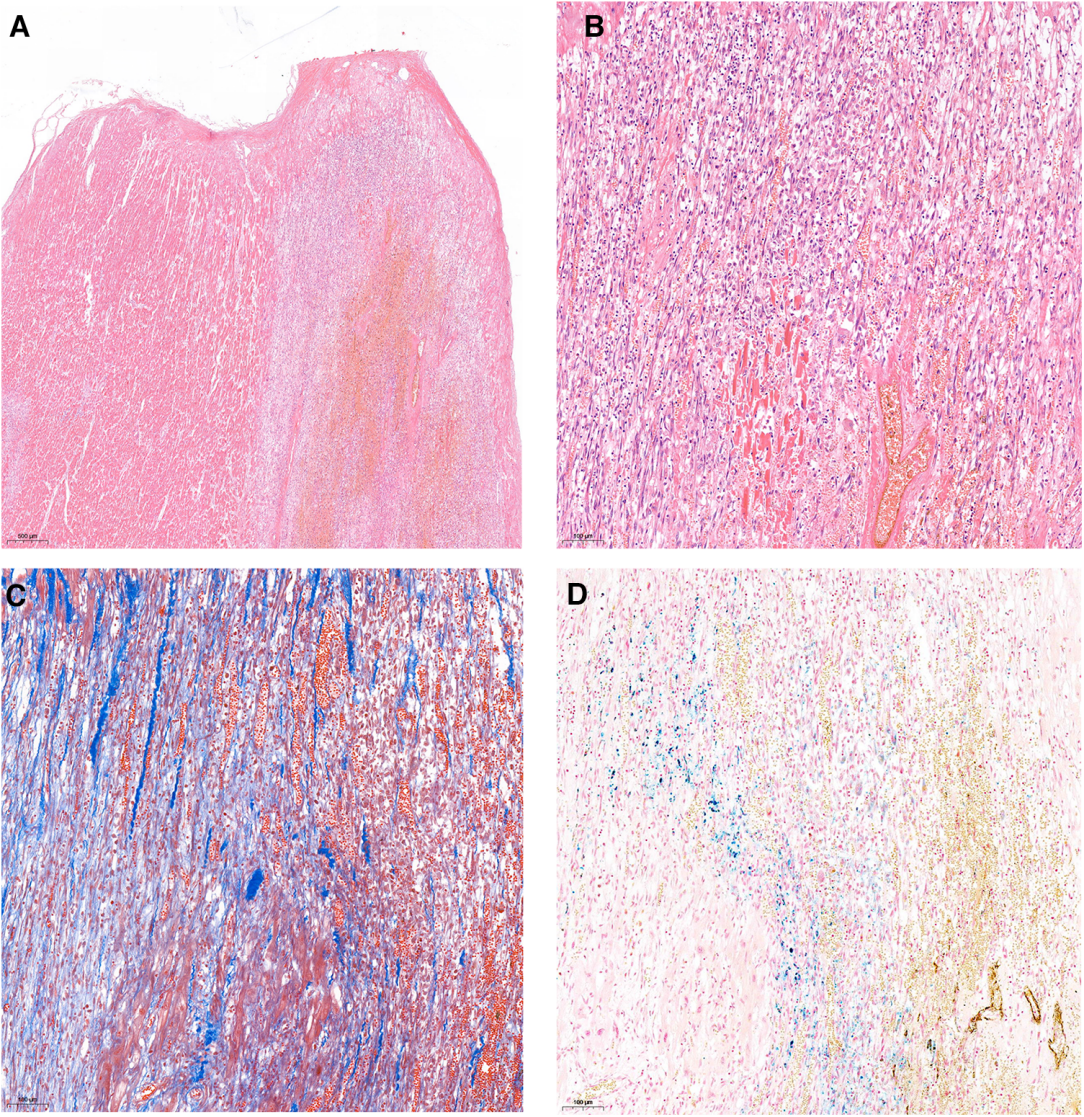
Histopathology examination of the trauma site on right ventricular anterior wall: thrombosis, degenerated and necrotic myocardium, and macrophages infiltration. (**A**) H&E staining, 20×; (**B**) H&E staining, 100×; (**C**) Masson's trichrome staining 100×, (**D**), Prussian blue staining, 100×.

Numerous multinucleated cells infiltrated the epicardium at the trauma site in the right ventricle. Thrombosis, degenerated and necrotic myocardium, and macrophage infiltration were also observed at the trauma sites. To further evaluate the time interval post-injury, Masson's trichome and Prussian blue staining were performed. Large amounts of blue collagen fibers distributed at the injury site were observed by Masson staining (Figure 1). Other organs showed anemia without any pathological changes.

## Discussion

The incidence of penetrating chest trauma has increased in urban regions in the past three decades. The cause of penetrating cardiac injuries varies according to the population and culture, and stabbing is still the leading cause in China (7, 8). Penetrating cardiac trauma is associated with immediate fatal outcomes; however, delayed cardiac rupture, although rare, can occur. To better understand the characteristics of such cases, we carefully reviewed the literature on delayed cardiac rupture in PubMed, and have summarized them in Table 1 (9–28). Delayed cardiac rupture can occur as soon as several hours post-trauma or as long as after 74 days, but most of them occur within a month, with stabbing accounting for half of the reported cases.

**Table 1:** Delayed cardiac rupture cases.

Authors and publication year	Age/Gender	Mechanism of injury	Admission CT scan	Interval time (days)	Primary injury location	Cardiac injury location	Outcome
Ochi et al., 2020 (9)	51/M	Stab	CT negative	28	Chest	Right ventricular-pericardial-pleural fistula	Well
Dokoupil et al., 2019 (10)	47/F	Accident	Rib fracture	34	Chest	Left ventricle rupture	Autopsy
Pooniya et al., 2016 (11)	2/M	Accident	None	7	Negative	Left ventricle rupture	Autopsy
Greene et al., 2016 (12)	21/F	Fall	CT: negative	10	Chest	Left ventricle rupture	Well
Esfahanizadeh et al., 2013 (13)	19/M	Stab	None	60	Chest	Aorto-RV Fistula	Well
Bartoloni et al., 2013 (14)	29/M	Stab	Chest x-ray and CT: left hemothorax	9	Chest	Coronary wall rupture	Autopsy
Ueda et al., 2011 (15)	75/F	Blunt chest trauma	Unknown	74	Chest	Left ventricular rupture	Well
Hermens et al., 2009 (16)	70/M	Accident	None	28	Chest	Hemorrhagic myocardial defect	Well
Babin-Ebell et al., 2008 (17)	25/M	Stab	Echocardiography	Sever hours	Chest	Ventral septal defect	Well
Eisenman et al., 2006 (18)	18/M	Stab	None	30	Chest	Ventricular puncture	Well
Moore et al., 2006 (19)	39/M	Stab	Chest radiograph and CT normal	21	Chest	Atriocaval laceration	Well
Murai et al., 2003 (20)	36/M	Fall	Echocardiography: negative	20 h	Chest	Right ventricular rupture	Autopsy
Murillo et al., 2002 (21)	10/M	Accident	CT: Haemo-pneumothorax	140 min	Chest	Left ventricular rupture	Autopsy
Lassus et al., 2001 (22)	44/M	Accident	x-ray, CT: negative	14	Chest	Right ventricle rupture	Well
Klingkenberg et al., 1994 (23)	34/F	Stab	Unknown	3	Chest	Right ventricle	Well
Pollak et al., 1991 (24)	7/M	Fall	None	8	Chest	Left ventricular rupture	Well
Martin et al., 1986 (25)	48/M	Stab	Unknown	42	Chest	Coronary arteriovenous fistula	Well
Lempinem et al., 1972 (26)	38/M	Stab	Unknown	9	Chest	Right ventricle	Well
Pastor et al., 1961 (27)	25/M	Stab	Unknown	35	Abdomen	Ventricle	Well
	31/M	Stab	Unknown	74	Negative	Negative	Well
Cosman et al., 1958 (28)	15/M	Stab	x-ray: right hemothorax	5	Chest	Right auricular	Well

Table 1 shows that according to previous reports, the left ventricle is the most common site of injury with delayed cardiac rupture. However, the anatomical location of the heart causes the right ventricle to be most frequently affected by a thoracic stab injury (29). The thinner wall of the right ventricle makes it impossible to close the defect by muscle overlap and contraction. Hence, the penetrating trauma may result in copious bleeding, even with low intraventricular pressure, resulting in rapid death. By contrast, the thick muscular wall in the left ventricle may easily close a stab injury and thus seal the trauma. However, even if the persistent bleeding is slight, it may still eventually result in cardiac tamponade, even with drainage intervention. The atria are most susceptible to penetrating trauma, as they completely lack a sealing effect (30). The formed thrombosis, which adheres tightly to the wound as in Case 1 may be the origin of the delayed cardiac rupture.

The data in Table 1 indicate that non-penetrating chest trauma can cause cardiac rupture, and common blunt cardiac rupture injuries among civilians, including traffic accidents, falls, heavy impact, and even a punch, can cause fatal damage. Cardiac ruptures are not always accompanied by thoracic wall injuries or rib fractures (31). Delayed cardiac rupture is often associated with hemopericardium as bleeding into the pericardial sac. To better understand the fatal outcome of delayed chest trauma, we searched for delayed hemopericardium or cardiac tamponade in PubMed, and have summarized the literatures on negative or healed cardiac injuries in Table 2 (32–44).

**Table 2:** Delayed hemopericardium or cardiac tamponade cases.

Authors and publication year	Age/Gender	Mechanism of injury	Admission CT scan	Interval time (days)	Primary injury location	Cardiac injury location	Outcome
Almehmadi et al., 2016 (32)	23/M	Stab	Negative	10	Chest + right ventricle laceration	Negative	Well
Khidir et al., 2015 (33)	19/M	Fall	Chest x-ray: normal	12	Chest	Negative	Well
Donahoe et al., 2013 (34)	20/M	Stab	Echocardiography: effusion	6	Chest	Negative	Well
Kanchan et al., 2012 (35)	71/M	Fall	Chest radiograph: multiple fractures	8 h	Negative	Right ventricle contusion	Autopsy
Nijjer et al., 2010 (36)	21/M	Stab	Chest radiograph: normal	100	Chest	Negative	Well
Liang et al., 2009 (37)	58/M	Accident	CT: sternal fracture	14	Chest	Negative	Well
Harris et al., 2003 (38)	Median: 28 (range: 14–53) 23/M; 1/F	Stab	Unknown	3–31 median 14.2	Chest	Unknown	Well
Kelsey et al., 1999 (39)	21/M	Fall	None	7	Chest	Negative	Well
Mechem et al., 1997 (40)	35/M	Stab	Chest x-ray, echocardiogram: normal.	19	Chest	Pulmonary artery laceration	Well
Raney & Kennedy, 1997 (41)	28/M	Stab	Unknown	21	Chest	Negative	Well
Bellanger et al., 1996 (42)	35/M	Stab	Unknown	21	Chest	Negative	Well
Bowers et al., 1994 (43)	21 months/W	Fall	None	7	Chest	Negative	Well
Aaland et al., 1991 (44)	50/M	Stab	Unknown	14	Chest	Negative	Well

The clinical manifestations of chest traumas listed in Tables 1, 2 are common in that their condition is rather stable after primary medical intervention. Moreover, other examinations such as chest radiography and computed tomography show negative findings, even with cardiac rupture and effusion. A previous study has indicated that delayed cardiac ruptures can be asymptomatic (45). The post-trauma ECG may initially be abnormal; however, ECG changes are not specific, as they can also occur in normal autopsy findings, and delayed cardiac rupture may occur even with normal ECG post-injury (46–48). This was the same as the CK-MB level and echocardiography with respect to cardiac injury. CK-MB, echocardiography, repeated ECG, and chest radiographic examinations may play a role in suspected cardiac injury cases (22, 49).

In 2023, Sessa et al. reviewed the published studies focusing on penetrating cardiac injury associated with firearm from 1990 to 2022, and identified 38 articles, 39 cases were involved (6). Among which, 33 were males, the entrance wound is located in the anterior chest in 30 cases. Based on the study, he suggested that timely transport, resuscitation, and immediate surgery were the critical management in the therapy of penetrating cardiac injury. In 2022, Berrichi et al. reported delayed cardiac herniation after a traumatic pericardial rupture in an adult male who fall from 8m high, and the patient was rescued through timely surgery (50). In the present case, the entrance wound is on left front chest, and the decedent was given immediate surgery and he was recovered well at first. However, few days later he suffered from delayed cardiac rupture and tamponade and died. The asymptomatic of his later fatal complications delayed the diagnose of his complication and eventually resulted in his tragedy.

Although rare, delayed cardiac rupture and tamponade after chest trauma is a challenge in clinical and forensic practice. Physicians should bear in mind this rare complication to provide better medical therapy. In forensic practice, the original cause of later cardiac complications and time interval after the primary injury make the real link underlying them more complicated. Forensic pathologists should carefully document the primary injury and thoroughly investigate the patient's medical history and interval time through histopathological examination and other advanced technologies.

## Conclusion

Delayed cardiac rupture and tamponade after chest trauma are rare in clinical and forensic practice and may cause sudden death. The casual link between the primary injury and later complications remains to be elucidated.

## Data Availability

The original contributions presented in the study are included in the article/Supplementary Material, further inquiries can be directed to the corresponding author.
